# Impact of Fibroblast-Derived SPARC on Invasiveness of Colorectal Cancer Cells

**DOI:** 10.3390/cancers11101421

**Published:** 2019-09-24

**Authors:** Daniel Drev, Felix Harpain, Andrea Beer, Anton Stift, Elisabeth S. Gruber, Martin Klimpfinger, Sabine Thalhammer, Andrea Reti, Lukas Kenner, Michael Bergmann, Brigitte Marian

**Affiliations:** 1 Department of Medicine 1, Institute of Cancer Research, Medical University of Vienna, Borschkegasse 8a, 1090 Vienna, Austria; daniel.drev@meduniwien.ac.at (D.D.);; 2 Department of Surgery, Division of General Surgery, Medical University of Vienna, Währinger Gürtel 18–20, 1090 Vienna, Austria; felix.harpain@meduniwien.ac.at (F.H.); anton.stift@meduniwien.ac.at (A.S.); elisabeth.s.gruber@meduniwien.ac.at (E.S.G.); michael.bergmann@meduniwien.ac.at (M.B.); 3 Clinical Institute of Pathology, Medical University of Vienna, Währinger Gürtel 18–20, 1090 Vienna, Austria; andrea.beer@meduniwien.ac.at (A.B.); lukas.kenner@meduniwien.ac.at (L.K.); 4 Department of Pathology, Social Medical Center South-Kaiser Franz Josef Hospital, Kundratstraβe 3, 1100 Vienna, Austria; martin.klimpfinger@wienkav.at (M.K.); sabine.thalhammer@wienkav.at (S.T.)

**Keywords:** colorectal cancer, tumor microenvironment, cancer-associated fibroblasts, SPARC, 3D co-culture

## Abstract

Secreted protein acidic and rich in cysteine (SPARC) is a matricellular protein modulating cell-matrix interactions and was found up-regulated in tumor stroma. To explore the effect of high stromal SPARC on colorectal cancer (CRC) cell behavior and clinical outcome, this study determined SPARC expression in patients suffering from stage II and III CRC using a publicly available mRNA data set and immunohistochemistry of tissue microarray sections. Moreover, in vitro co-culture models using CRC cell lines together with colon-associated fibroblasts were established to determine the effect of fibroblast-derived SPARC on cancer cells. In 466 patient samples, high SPARC mRNA was associated with a shorter disease-free survival. In 99 patients of the tissue microarray cohort, high stromal SPARC in the primary tumor was an independent predictor of shorter survival in patients with relapse (27 cases; HR = 4574, *p* = 0.004). In CRC cell lines, SPARC suppressed phosphorylation of focal adhesion kinase and stimulated cell migration. Colon-associated fibroblasts increased migration velocity by 30% and doubled track-length in SPARC-dependent manner. In a 3D co-culture system, fibroblast-derived SPARC enhanced tumor cell invasion. Taken together, stromal SPARC had a pro-metastatic impact in vitro and was a characteristic of aggressive tumors with poor prognosis in CRC patients.

## 1. Introduction

Secreted protein acidic and rich in cysteine (SPARC) is a matricellular glycoprotein that is produced in many organs in processes of rapid proliferation or remodeling [1] and is essential for wound healing [2]. SPARC modulates cell behavior by binding to extracellular matrix (ECM) proteins and cell surface receptors, but no specific SPARC-receptor has been found. Therefore, the protein is presumed to inhibit cell-matrix interactions in a competitive manner [3]. The biological role of SPARC seems to be highly context-dependent, but matrix remodeling and counter-adhesive effects have been reported consistently (reviewed in [4]).

With regard to cancer development, SPARC has been described as a tumorsuppressor in some tumor types (e.g., in ovarian or bladder cancer [5,6]), while it was found overexpressed as a possible promoter of tumorigenesis in others (melanoma, breast cancer, and glioblastoma [7,8,9,10]).

In the human intestine, SPARC is detected at the epithelial-mesenchymal interface and in the mesenchyme in early development until mid-gestation. In the adult colonic mucosa, the protein is restricted to the muscularis mucosa, but is absent from the epithelium [11]. In colonic cancers SPARC has been identified as an upregulated gene in 7 expression profiling studies [12]. In an APCMin/+ mouse model, SPARC has been described as a pro-tumorigenic factor, because its deficiency suppressed tumorigenesis [13]. By contrast, it was identified as a tumor suppressor silenced by promoter methylation in human colorectal cancers (CRC), including CRC cell lines HCT116 and SW480 [14,15,16]. Our own recent tissue proteomics data offer to consolidate the contradiction, as we found increased abundance of SPARC protein in CRC as compared to normal tissue, but the protein was almost exclusively located in the tumor stroma [17].

To further explore the role of SPARC in CRC, this current study analyzed the relationship of stromal SPARC expression to clinical parameters in CRC patients using data of stage II and III patients in a publicly available mRNA expression data set [18] and in a tissue microarray consisting of 99 patient samples. The role of SPARC in CRC cell migration and invasion was investigated in standard and 3D co-culture models.

## 2. Results

### 2.1. Impact of SPARC on CRC Outcome

**Figure 1:**
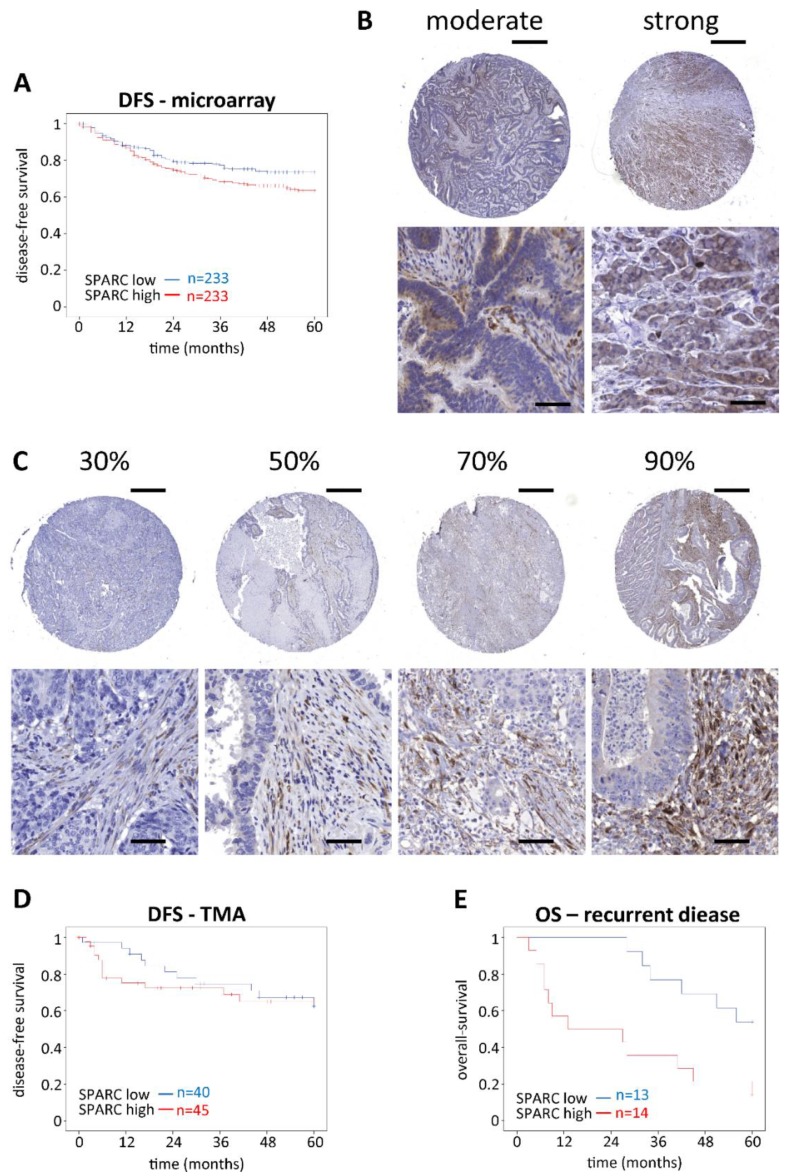
Impact of SPARC expression/abundance on OS and DFS. (**A**) Kaplan-Meier curves were plotted by choosing the cutoff at the median SPARC mRNA expression (microarray, *n* = 466). Patients with high SPARC expression had a decreased 5-year DFS (*p* = 0.048). (**B**,**C**) Tissue microarray slides were stained with monoclonal anti-SPARC antibodies, scanned as described in materials and methods, and scored for positively stained cells in epithelium and stroma separately. The panels show representative examples for positive epithelium (**B**) and different levels of SPARC-positive stroma (**C**). Scale bars for the overview pictures (upper right corner) represent 500 µm. Scale bars for the zoomed images represent 50 µm. (**D**,**E**) For Kaplan-Meier analysis, the cut-off was again set at the median of positive stromal SPARC %. Stromal SPARC abundance did not affect 5-year DFS (**D**) (*n* = 85). Patients with disease recurrence (*n* = 27) had a reduced OS when their primary tumors were highly positive for stromal SPARC (**E**) (*p* = 0.007). All statistical analyses were performed by using Log Rank tests. OS, overall-survival. DFS, disease-free survival. TMA, tissue microarray.

To investigate the impact of SPARC expression on CRC, we analyzed the gene expression of 466 human patients who suffered from stage II or stage III CRC, summarized in the public available dataset GSE39582 [18]. Analyzing Kaplan-Meier plots, there was no significant impact on the 5-year overall survival (OS) based on SPARC expression (Supplementary Figure S1A), but a markedly decreased 5-year disease free survival (DFS) of patients with high compared to low SPARC expressing tumors (Figure 1A). Gender, age, tumor stage, chemotherapy, location and SPARC expression were assessed using univariate Cox regression analysis to identify their impact on DFS (Table 1). A statistically significant effect was found for tumor stage, chemotherapy, and SPARC expression. Furthermore, in the multivariate analysis it was shown, that tumor stage was the main predictor for DFS and SPARC expression just failed significance with *p* = 0.051.

In addition, we analyzed SPARC protein abundance of CRC tissue from 99 patients with immunohistochemistry (IHC). The patients’ median age was 76 years (range 54–97 years); 40 patients were female and 59 male and DFS data were available for 85 patients. The median follow-up period was 46 months (range 0–173 months). Only 16/99 specimen had SPARC-positive tumor epithelium (Figure 1B) while the vast majority was negative. By contrast, all specimens were SPARC-positive in their stroma with a median SPARC score of 45% of all stromal cells (range 4.5–86.7%) (Figure 1C). There was no correlation between SPARC positive tumor epithelium and survival (data not shown). Similar to the mRNA microarray, there was no effect of stromal SPARC protein levels on the 5-year OS (Supplementary Figure S1B). Furthermore, there was no significant impact on the 5-year DFS, but within the first year after tumor resection, a notable shift towards shorter DFS was seen for tumors with high stromal SPARC abundance (*p* = 0.034, Log Rank test) (Figure 1D). This difference leveled out over 5 years, but it prompted us to look for additional evidence of aggressive tumors in relation to high stromal SPARC expression. For this, we analyzed OS in those 27 patients who suffered from relapse. They mostly received 5-FU-based combination therapy and developed metastasis predominately in the liver or in the lung. SPARC expression in the primary tumors negatively affected the 5-year OS (*p* = 0.007, Log Rank test) (Figure 1E). According to univariate Cox regression model age, location as well as high/low SPARC abundance were found to impact the 5-year OS (Table 2).

The multivariate analysis showed that location and high/low SPARC abundance were independent predictors to affect the 5-year survival of patients with relapse. The staining scores and clinical data for all patients can be found in Table S1 in the supplementary material. Additional information about the recurrent patients including exact tumor and recurrence location, as well as treatment regimens is summarized in Table S2 in the Supplementary Materials.

### 2.2. SPARC Expression in Cancer-Associated Fibroblasts 

**Figure 2:**
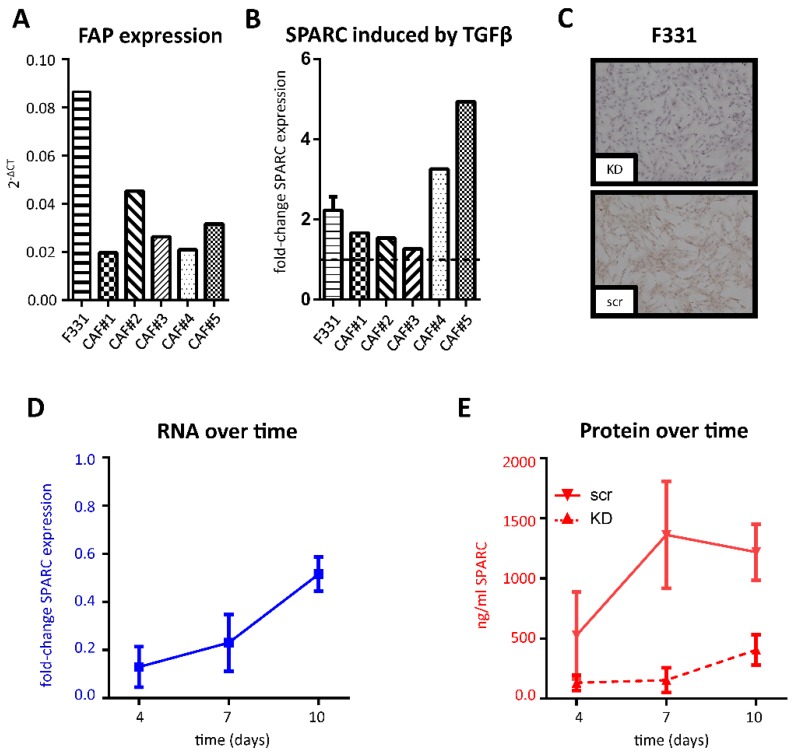
Fibroblast-derived SPARC: RNA was isolated from semiconfluent fibroblast cultures and FAP mRNA was analyzed by qRT-PCR relative to GAPDH as the housekeeping gene (**A**). F331 and CAFs were treated with 5 ng/mL TGFβ for a total of 72 h. SPARC mRNA expression was plotted as fold change to negative control and shown for F331 fibroblasts (*n* = 3) and for CAFs (single experiments); numbering of CAFs indicate the respective patient from whom the fibroblasts were isolated (**B**). In addition, F331 cells transfected with SPARC siRNA or scr control were grown on glass slides and stained by IHC with a SPARC specific mAB after 72 h (**C**); size bar = 100 µm. Knockdown efficiency in F331 cells was monitored over a total of 10 days (**D**) SPARC mRNA expression after siRNA transfection was calculated as fold-change ratio of KD to scr (*n* = 3). (**E**) SPARC protein levels secreted into the medium supernatant were evaluated by ELISA and plotted for both scr (solid line) and KD (dashed line) (*n* = 2). All data are pooled from experiments using 2 different siRNAs and given as mean ± SD. KD, knockdown; scr, scrambled control; siRNA, small interfering RNA.

To analyze the potential production and impact of stromal SPARC in vitro, fibroblasts were either isolated from fetal human colon (F331) [19] or human CRC tissue (cancer-associated fibroblasts (CAFs)). The isolated cell populations expressed fibroblast activated protein (FAP) (Figure 2A), but lacked the endothelial marker CD31 and keratin 20 (KRT20), a marker of colorectal adenocarcinoma cells (Figure S2), which demonstrates their stromal origin and activated status. SPARC mRNA was expressed by all fibroblast populations isolated, while expression in the used cancer cells was extremely low or absent (Figure S2D). Previous studies showed that SPARC expression can be induced by TGFβ in various cell types [20,21,22,23] and CRC cells have been demonstrated to secrete high levels of TGFβ [24]. Furthermore, our own previous study identified SPARC to be part of a TGFβ-dependent protein signature by pathway analysis [17]. Therefore, we stimulated fibroblasts with recombinant TGFβ for 72 h, which resulted in an induction of SPARC mRNA levels in all populations of CAFs as well as in F331 (Figure 2B). Based on F331’s similar characteristics and their easier availability they were used as model cells for most of the in vitro studies. To monitor the effects of fibroblast derived SPARC, we performed knock down (KD) experiments in F331 and visualized intracellular SPARC in F331 plated on glass slides and transfected with either SPARC small interfering RNA (siRNA) (KD) or scrambled (scr) control (Figure 2C).

Moreover, we measured both mRNA and secreted SPARC protein levels in the medium supernatant over a total of 10 days (Figure 2D). SPARC mRNA levels increased over time, but were still down to approximately 50% of the scr control 10 days after KD. SPARC protein levels reached a maximum after 7 days (1.36 ± 0.45 µg/mL) in the control group compared to 0.15 ± 0.1 µg/mL in KD fibroblasts. 10 days after KD, differences between the two groups began to level out, but were still down to about 1/3 of the control group (1.22 ± 0.23 µg/mL for scr compared to 0.41 ± 0.13 µg/mL for KD).

### 2.3. SPARC and Tumor Cell Migration

**Figure 3:**
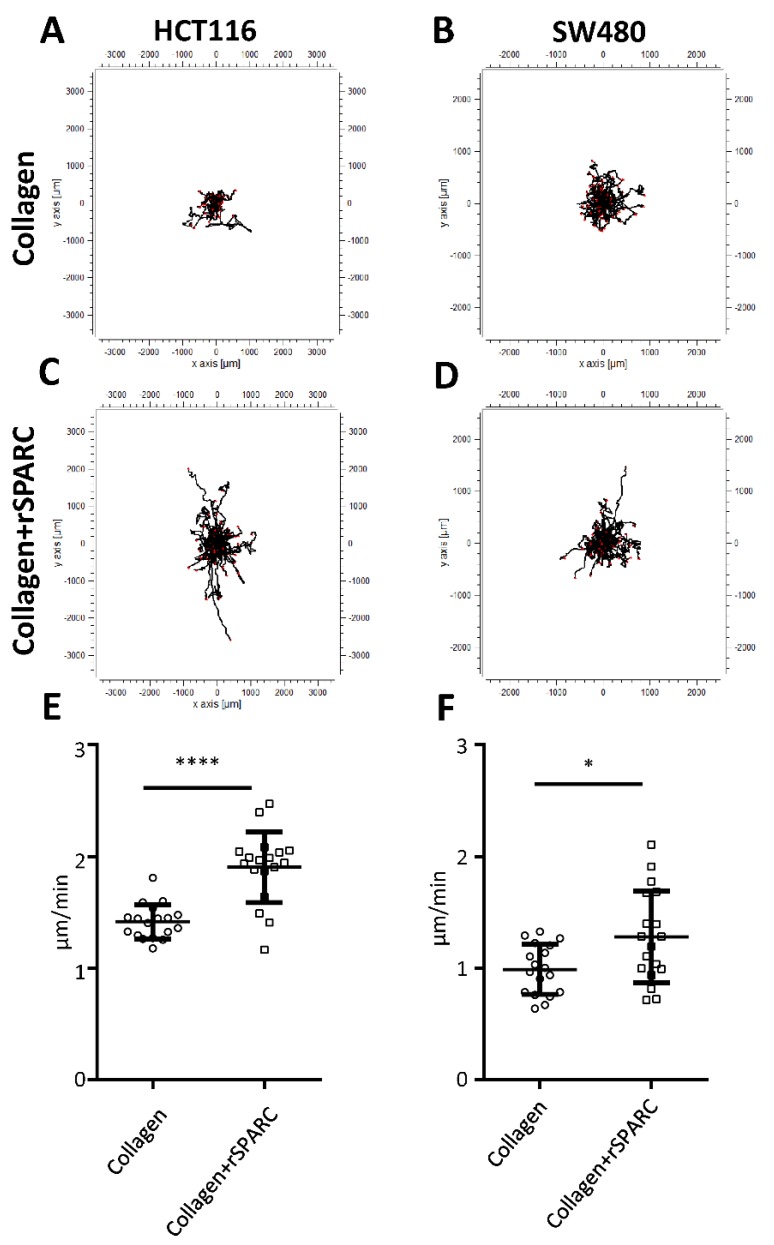
Influence of rSPARC on cancer cell migration. Cancer cells were seeded on collagen I coated plates supplemented with either PBS or PBS + rSPARC and tracked for a total of 22 h. Tracks were transformed to have the same starting points, and HCT116 as well as SW480 cells migrating on either collagen (**A**,**B**) or collagen + rSPARC (**C**,**D**) were plotted. Average migration speed in µm/min are shown for each well (n = 18) for a total of 3 independent experiments for HCT116 (**E**) and SW480 (**F**); statistical analyses were performed by using the Mann-Whitney test. *: *p* < 0.05; ****: *p* < 0.0001. Scatter dot plots: center line, means; whiskers, SD; rSPARC, human recombinant SPARC; PBS, phosphate buffered saline.

As SPARC has been described as a modulator of cell-matrix interactions [4], tumor cells were plated on culture substrates consisting of type I collagen with or without rSPARC. Tumor cell models were HCT116 and SW480 CRC cell lines. They were tracked using CellTracker and their migration analyzed using life cell microscopy for 22 h. Plots deducted from the resulting videos depict the migration tracks and typical examples for HCT116 and SW480 cells are shown in Figure 3. On collagen alone, migration paths were well within a radius of 1000 µm (Figure 3A,B), while addition of SPARC induced a multitude of longer paths (Figure 3C,D). Velocity was higher for HCT116 cells (1.42 ± 0.15 µm/min, Figure 3E) as compared to SW480 (0.99 ± 0.22 µm/min, Figure 3F). For both cell lines, the addition of rSPARC resulted in a statistically significant increase in migration velocity (Figure 3E,F).

### 2.4. SPARC Decreased Interaction with the Collagen Matrix

**Figure 4:**
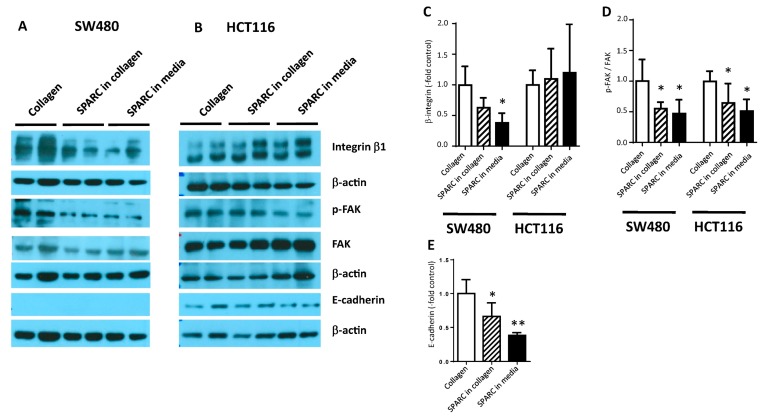
SPARC-induced molecular changes. SW480 (**A**) and HCT116 (**B**) cells were seeded on collagen I. 750 ng/mL SPARC were added to the collagen gel or the culture medium. 24 h later protein lysates were prepared and analyzed by western blotting using antibodies recognizing integrin β1, FAK, p-FAK and E-cadherin. β–actin was used as loading control. Band intensity was measured using ImageJ software and quantification results of at least 3 independent cultures were pooled. (**C**) integrin β1, (**D**) relative phosphorylation of FAK, (**E**) E-cadherin in HCT116. * different from control at *p* < 0.05. FAK, focal adhesion kinase; p-FAK, phosphorylated FAK.

To analyze tumor cell–ECM interaction, cells were plated on type I collagen in the presence or absence of SPARC for 24 h. SW480 cells had decreased levels of integrin β1, as well as focal adhesion kinase (FAK) and phosphorylated FAK (p-FAK) when they were plated on a SPARC-containing matrix or exposed to SPARC in the culture supernatant (Figure 4A,C,D). In HCT116 cultures, no impact of SPARC on integrin β1 could be observed and FAK protein was even increased in the SPARC groups (Figure 4B,C). Phosphorylation of FAK decreased, however, indicating down-modulation of integrin/FAK-dependent signaling (Figure 4B,D). While E-cadherin was below detection level in SW480 cells (Figure 4A), it was found downregulated in HCT116 (Figure 4B,E).

Since rSPARC deposited in a Collagen I matrix increased the mobility of cancer cells, we wanted to investigate the effects of fibroblast derived SPARC on cancer cell motility in a more complex system. In a first step, F331 cells were grown to form dense monolayers and secrete SPARC into their culture medium and deposited matrix. Afterwards, cancer cells were seeded on top of these 2D fibroblast layers and migration was analyzed by live cell microscopy to determine track length and migration velocity.

**Figure 5:**
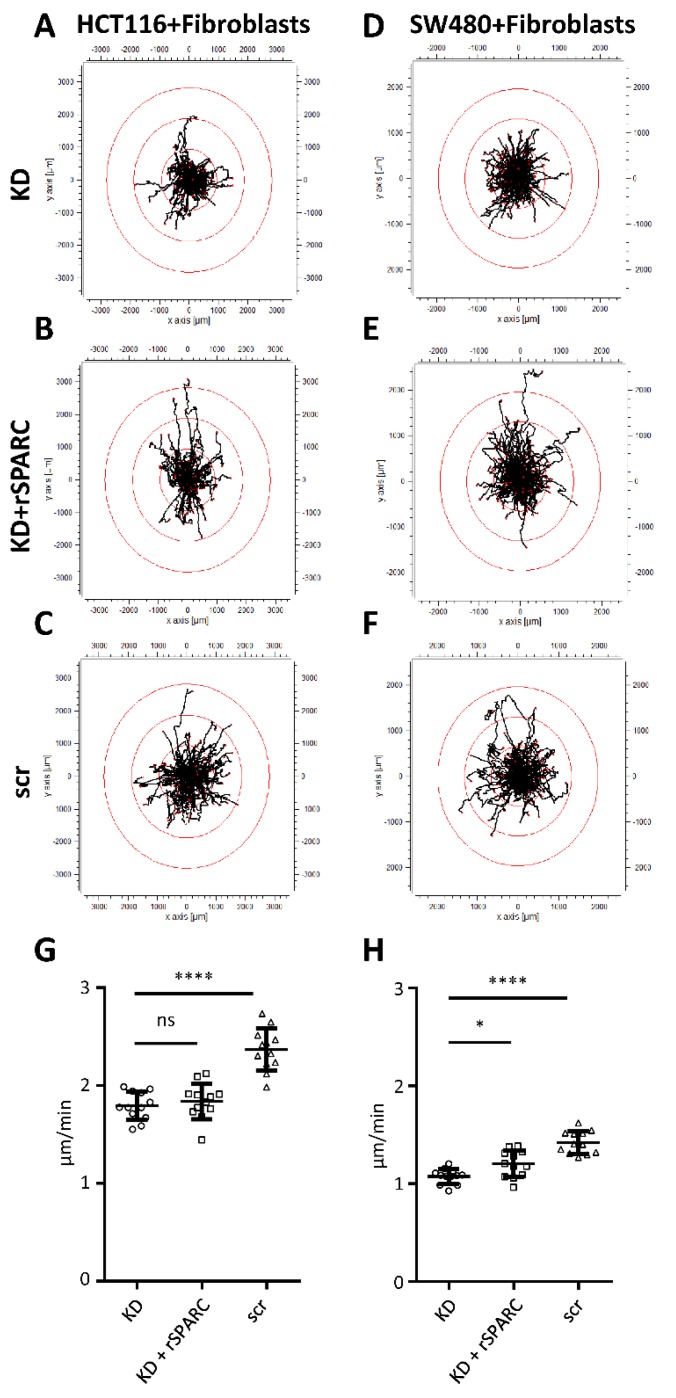
Influence of fibroblast derived SPARC on cancer cell migration. F331 fibroblasts transfected with either scr control, SPARC siRNA (KD) or KD cells with additional rSPARC were seeded at high numbers and allowed to secrete and modulate their respective ECM. After 5 days, cancer cells were added on top of the fibroblast monolayers and migration of individual cells was recorded for a total of 22 h. (**A**–**C**) shows the resulting tracks for HCT116 cells migrating on F331 with KD, KD + rSPARC and scr control. Similar tracks for SW480 cells are summarized in (**D**–**F**). Red circles indicate track length based on maximum Euclidian distance and were positioned at 50%, 100% and 150% of the longest track of the KD group. The average migration speed in µm/min of HCT116 (**G**) and SW480 (**H**) was plotted as mean values for each well (*n* = 10) of 2 independent experiments using 2 different siRNAs. Statistical analysis for different migration velocities was performed by using the Kruskal-Wallis test followed by Dunn’s multiple comparisons test. *: *p* < 0.05; ****: *p* < 0.0001. Scatter dot plots: center line, means; whiskers, SD; KD, knockdown; rSPARC, human recombinant SPARC; scr, scrambled control.

For HCT116 cells on fibroblasts with SPARC KD only 16.5% of the tracks extending from the migration origin were longer than the applied cutoff for discrimination of track length (Figure 5A). Tracks above threshold increased after addition of rSPARC both in number (30.1%) and track length (Figure 5B). Control fibroblasts producing normal levels of SPARC were the substrate, which induced the highest number of elongated tracks compared to the other 2 groups (40.1%, Figure 5C). For SW480 cells, migration on F331 fibroblasts with SPARC KD resulted in 20.4% of tracks above the threshold, while supplementing fibroblasts with rSPARC or transfected with scr control increased the number of long tracks to 34.1% and 33.3%, respectively (Figure 5D–F). Analysis of migration velocity mostly supported the same conclusion: in the scr controls, velocity was higher than in the respective KD groups (2.37 ± 0.21 µm/min vs. 1.79 ± 0.15 and 1.42 ± 0.12 µm/min vs. 1.07 ± 0.08 for HCT116 and SW480, respectively) (Figure 5G,H). For SW480 cells, rescue by addition of rSPARC was also reflected by a significant increase of migration velocity as compared to the KD group (Figure 5H). For HCT116 cultures, velocity remained at the KD level (Figure 5G) despite the recognizable presence of longer tracks (Figure 5B).

### 2.5. SPARC and Tumor Cell Invasion

To study the impact of stromal SPARC on tumor cell invasion, connective tissue reconstructs were produced by suspending fibroblasts in a matrix consisting of type I collagen and methylcellulose (see Figure S3A for an overview). Invasion depth was measured from tissue sections stained by IHC with an anti-cytokeratin antibody (Figure S3B). 

There was no invasion in the absence of fibroblasts (Figure S3C (1) and (2)). For invasion assays, src control and SPARC KD fibroblasts were used and SPARC expression after 10 days was still reduced in the KD group (Figure S3C (3) and (4)).

**Figure 6:**
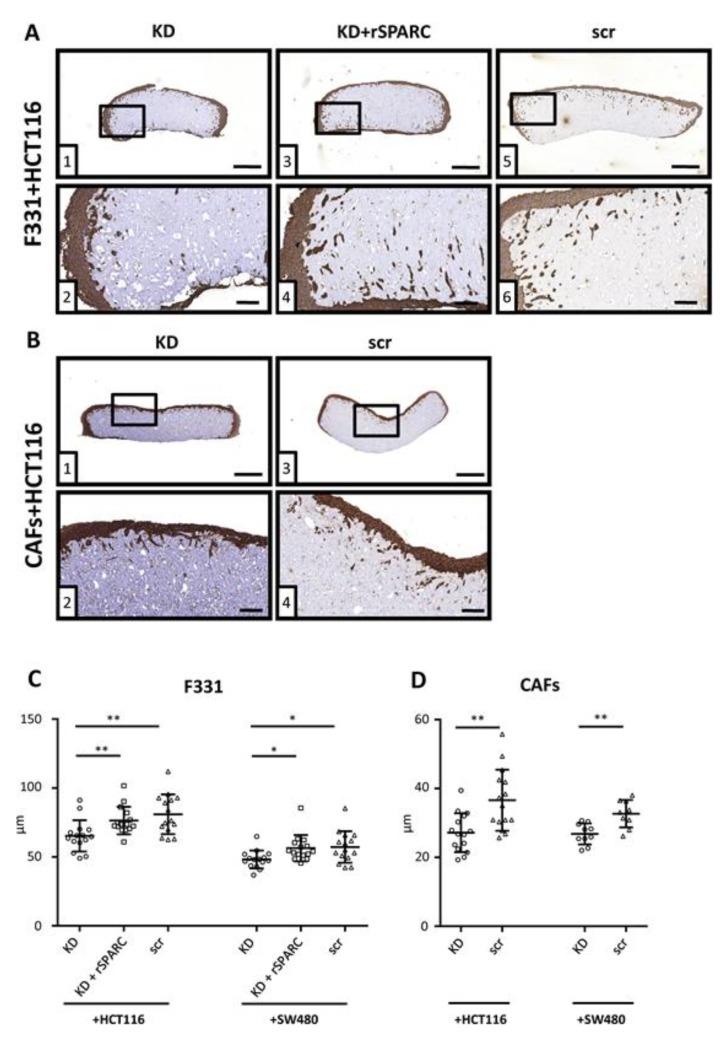
3D co-culture tissue reconstructs F331 fibroblasts with either SPARC KD, KD + rSPARC or scr control were suspended into a 3D matrix consisting of methylcellulose + collagen type I and allowed to grow and structure their 3D matrix for 4 days. Cancer cells were seeded onto these reconstructs, incubated for an additional 6 days and IHC stained with a cytokeratin antibody. Typical sections of either F331 (**A**) or CAFs (**B**) co-cultured with HCT116 cells used for quantification. Overview of sections can be viewed in corresponding upper panels (size bar = 500 µm) and zoomed images in lower panels (size bar = 100 µm), respectively. For each reconstruct, 5 sections separated by 100µm were analyzed. Results are shown as average invasion depth per section (*n* = 15) for 3 independent experiments using 2 different siRNAs for both HCT116 and SW480 (**C**). In addition, reconstructs with CAFs co-cultured with either HCT116 or SW480 (**D**) were evaluated as well (15 sections, 3 independent experiments for HCT116 and 10 sections, 2 independent experiments, for SW480; 2 different siRNAs for both cell lines). Statistical analysis to evaluate differences between 3 groups were performed by using the Kruskal Wallis test followed by Dunn’s multiple comparisons test, and the Mann Whitney test for 2 groups. *: *p* < 0.05; **: *p* < 0.01; Scatter dot plots: center line, means; whiskers, SD; KD, knockdown; rSPARC, human recombinant SPARC; scr, scrambled control.

Typical examples of IHC sections used for the analysis of cancer cell invasiveness are shown for HCT116 co-cultured with F331 (Figure 6A) and CAFs (Figure 6B). Similar images of SW480 cells can be found in Figure S4. Average invasion depth was 80.96 ± 14.45 µm for HCT116 scr control cells and 57.24 ± 11.47 µm for SW480 scr controls (Figure 6C). SPARC depletion inhibited invasiveness in both cell lines as shown by a reduction of invasion depth by about 20% to 65.28 ± 11.38 µm and 48.16 ± 6.51 µm respectively, while this effect could be at least partly rescued by addition of rSPARC into the 3D matrix. When primary CAFs were used to produce the reconstituted stroma, differences in invasion depth between scr and KD groups were 36.61 ± 8.88 µm vs. 27.18 ± 5.64 µm for HCT116 and 32.69 ± 4.00 µm vs. 26.84 ± 3.06 µm for SW480 cells (Figure 6D).

## 3. Discussion

SPARC has been found up-regulated in colorectal tumors [17,25] and was identified as one of the genes that specifically discriminated between adenomas and carcinomas [12,26]. Despite this, the actual role of SPARC in CRC biology is still controversial, as the biological impact of SPARC protein is highly context-dependent and differs based on the cellular origin of the protein. In CRC the protein is silenced as a tumorsuppressor in the cancer cells [14,16], while it is predominantly expressed in the tumor stroma [17,25] where it is part of a TGFβ-associated protein signature [17].

Our analysis of clinical outcome focused on patients diagnosed at stage II or III and used mRNA expression data from a publicly available data set [18] as well as protein abundance results obtained from patients recruited at the General Hospital in Vienna. Neither data set identified SPARC as an indicator of good outcome as published previously [25,27]. By contrast, high-SPARC tumors resulted in a shorter DFS in the mRNA-based GSE39582 dataset. Of the 99 patients of the Vienna cohort, all expressed SPARC in the tumor stroma, but only 16 contained SPARC-positive cancer cells. Due to the low number of patients we cannot draw any conclusions about tumor cell-derived SPARC and focused our study on stromal SPARC.

In that regard, there was no overall impact of SPARC on 5-year DFS. However, patients with high stromal SPARC were more prone to early recurrence: of the 27 patients who suffered relapse, 12 had a DFS of <12 months and 10 of these were in the high stromal SPARC group. While this narrowly missed statistical significance, it is a striking observation that should be further explored in a larger cohort. In addition, for those patients who did suffer a relapse, high stromal SPARC was a strong predictor of shorter OS: of the 27 patients with recurrent disease, 10 had an OS <30 months and 9 of those were both in the early recurrence group and in the high SPARC group. In this subset of patients, high SPARC abundance was a predictor of survival independent of stage, therapy and tumor location. Together, the clinical results from our study point to stromal SPARC as an indicator of more aggressive tumors and worse outcome, which differs widely from previous reports on SPARC in CRC [25,27] that reported SPARC as a positive prognostic marker. This may be due to ethnic differences between the cohorts studied or different adjuvant treatment—e.g., Chew et al. recruited their patients as early as 1996–2002 and adjuvant treatment regimens have greatly improved since then. This may be of relevance, because SPARC has been shown to modulate treatment response in ovarian cancer [28] and interference with sensitivity to radiation and drug exposure in CRC cell lines [29].

Our analysis of the clinical data is in line with the results from the cell line co-culture models demonstrating increased tumor cell aggressiveness in the presence of SPARC. Specifically, we have shown that SPARC deposited in the tumor stroma is an essential factor in tumor cell migration and invasion. Both fetal-derived F331 fibroblasts and primary CAFs produced and secreted SPARC and were further stimulated to do so by TGFβ. Addition of rSPARC to collagen matrices stimulated tumor cell migration. On the molecular level, SPARC weakened cell attachment to collagen: in SW480 cells, exposure to SPARC downregulated expression of integrin β1—a constituent of the collagen receptor (α2β1) as well as the fibronectin receptor (α5β1). Integrin β1 was chosen for the analysis, because it is an essential part of the collagen receptor [30]. Even though diverse roles for the integrin have been described in cancer [31], we have previously observed suppression of integrin β1 induced by 12(S)-lipoxygenase and its pro-inflammatory product 12(S)-hydroxyeicosatetraenic acid. At the same time, tumor cell migration and invasiveness of CRC cell line models were enhanced [32]. In HCT116 cells, no alteration of the integrin β1 level could be observed. Phosphorylation of FAK was reduced by SPARC in both cell lines. This seems to contradict reports that increased p-FAK enhanced metastasis by enabling interaction with changing matrix components and providing survival signaling for invading cancer cells [33]. With regard to SPARC, induction of pFAK formation was observed in glioma and melanoma [34,35], while decreased formation of pFAK was reported in ovarian cancer [36]. However, all these observations are derived from whole tumor tissue, while our results are derived from a cell culture model that provides collagen I as the only substrate for cell attachment. In this simple context decreased activation of FAK indicates decreased interaction with the collagen substrate, and no further conclusions about the role of FAK can be drawn [30]. The differences in SPARC-induced molecular alterations can be explained by the different molecular characteristics of the two cell lines. While SW480 carries the characteristics of mismatch-proficient CRC lines with mutations in APC, K-ras and p53 and loss of heterozygosity at chromosomal locations of tumor suppressor genes [37,38], HCT116 cells are mismatch-repair deficient and carry mutations in β-catenin and the TGFβ receptor [38]. In our hands an important difference was the expression of E-cadherin that was below detection level in SW480 indicating a mesenchymal phenotype, but was detectable in HCT116 and down modulated by exposure to SPARC.

Migration velocity was highest when the SPARC-containing matrix was deposited by fibroblasts. Tumor cells plated on dense monolayers of these fibroblasts migrated in a directed manner at velocities of 2.4 µm/min for HCT116 and 1.5 µm/min for SW480. Depletion of SPARC strongly suppressed migration activity and admixture of rSPARC partially rescued the effect, which has also been described for ovarian cancer [28]. Rescue of migration velocity by rSPARC was less efficient for HCT116 than for SW480 cells, indicating a different SPARC-based mechanism influencing migration speed. This was also evident from the different regulation of integrin β1 in the two cell lines. 

It has been previously shown by filter migration assays, that gastric cancer cells lost most of their invasive capacity when SPARC was knocked-down [39]. Here, we showed alterations in cancer cell invasion based on SPARC levels of fibroblasts in 3D co-culture. Tumor cell invasion into collagen matrices was enabled by fibroblasts structuring the matrix into tissue reconstructs. Again, SPARC-dependency was demonstrated by SPARC-depletion of the fibroblasts and rescue by rSPARC addition to the collagen gel. F331 fibroblasts and primary CAFs displayed qualitatively similar effects and the magnitude of the activity correlated with SPARC expression in the culture.

No SPARC-induced alterations have yet been found in the cancer cells that may explain the increased invasiveness. Therefore, the full picture of SPARC-mediated molecular effects and their impact on CRC progression is as yet incomplete. Ongoing studies could address the role of non-coding RNAs in SPARC-mediated mechanisms as they have been associated with CRC development [40,41]. Indeed, miR-211 (micro RNA-211) has been shown to down-regulate SPARC in hepatocellular carcinoma [42]. Alternatively, the crucial alterations may be in the fibroblast structured matrix that need to be further elucidated.

## 4. Materials and Methods 

### 4.1. Tissue Acquisition

This study was approved by the Ethics commission of the Medical University of Vienna (EK 1659/2012) and of the City of Vienna (EK 14-270-0215) and all participants were asked for their informed consent. Samples of colorectal carcinomas were obtained from patients suffering from CRC and underwent surgery at the General Hospital or the Kaiser Franz Josef Hospital in Vienna. For isolation of fibroblasts, tissue specimens were put in transport medium consisting of minimal essential medium (MEM) supplemented with 2% fetal calf serum (FCS), 100 U/mL Penicillin (Sigma-Aldrich, St. Louis, MO, USA), 0.1 mg/mL Streptomycin (Sigma-Aldrich), 0.1 mg/mL Kanamycin (Sigma-Aldrich) and 250 U/mL Nystatin (Sigma-Aldrich) and cooled on ice for transport. 

### 4.2. Analysis of Gene-Expression Microarray and Tissue Microarray

The publicly available data set GSE39582 [18] was processed using Excel to obtain a machine-readable format and included 466 patients suffering from AJCC/UICC stage II or III CRC. 

Tissue microarray comprised 99 patients suffering from AJCC/UICC stage II or III CRC that underwent resection at the Division of General Surgery, Medical University of Vienna between 2001 and 2012 (vast majority of 95 patients between 2008 and 2010). For each patient, only the primary tumors were included in this study. Slides were IHC stained with SPARC mAB (D10F10, Cell Signaling, Cambridge, UK) and the abundance of SPARC-positive cells scored in epithelium and stroma separately. Evaluation was done by three different blinded persons and differences in assessment resolved by discussion. 

Events of OS and DFS were normalized to a maximum of 5 years. As cutoff for all survival-based analysis, the median SPARC abundance was chosen. OS, DFS and univariate as well as multivariate cox regression analysis were performed with IBM SPSS Statistics for Windows, Version 24.0 (IBM) software. For Kaplan-Meier curves, Log Rank tests were performed and found to be significant if *p* < 0.05.

### 4.3. Cell Culture

All cells were cultivated in DMEM supplemented with 10% FCS at 37 °C in a humidified chamber containing 7.5% CO_2_. F331 fibroblasts [19] were used until passage 20 and CAFs for a maximum of 8 passages. CRC cell lines HCT116 and SW480 were obtained from the ATCC. All cells were regularly checked for mycoplasma contamination and were authenticated by Eurofins Genomics.

### 4.4. Isolation of Cancer-Associated Fibroblasts (CAF)

Tissue specimen were mechanically dissected, washed extensively in washing medium (MEM plus 2% FCS and penicillin/streptomycin) including incubations in 0.75% hypochlorite for 5 min and in 70% ethanol for 30 s. Next, tissue samples were cut in small pieces, immersed in Dulbeccos’s minimal essential medium (DMEM) supplemented with 312.5 U collagenase II/0.1 g tissue (Invitrogen, Carlsbad, CA, USA) in 5 mL MEM and incubated at 37 °C under constant shaking. Finally, digested tissues were poured through a cell strainer (BD Falcon, Franklin Lakes, NJ, USA) with appropriate mesh (size 70–100 µm, depending on grade of digestion) and the resulting cell suspension plated onto petri dishes in DMEM supplemented with 10%FCS, 100 U/mL Penicillin, 0.1 mg/mL Streptomycin, 0.1 mg/mL Kanamycin and 250 U/mL Nystatin.

### 4.5. Transforming Growth Factor Beta (TGFβ) Stimulation Assay

Fibroblasts were seeded in DMEM with 10% FCS at a density of 2 × 10^5^ cells per well in 6 well plates supplemented either with 5 ng/mL human TGFβ1 (Peprotech, Rocky Hill, NJ, USA) or the same volume of PBS. After 72 h cells were lysed using Trifast (Peqlab, Erlangen, Germany) followed by RNA isolation, subsequent reverse transcription and qRT-PCR.

### 4.6. RNA Isolation and Quantitative Real-Time PCR

RNA was isolated using Trifast reagent (Peqlab) according to manufacturer’s instructions and consequently transcribed into cDNA. Quantitative real-time PCR was carried out using the ABI 7500 fast real-time PCR system (Applied Biosystems, Foster City, CA, USA) as previously described [31] in combination with either Taqman kits (Thermo Fisher Scientific, Waltham, MA, USA) for GAPDH (HS00266705_g1), SPARC (HS00239160_m1), FAP (HS00990791_m1) and Keratin20 (Hs0000300643_m1) or Sybr green-based primer pairs for GAPDH (FW: CGAGATCCCTCCAAAATCAA; RW: AGAGATGATGACCCTTTTGG) and CD31 (FW: CATTACGGTCACAATGACGA; RW: CTTGAACTTCCGTGTACTGC).

### 4.7. Gene Expression Knock-down

SPARC was knocked down by transfection with stealth sirna (either hss110131 or hss110132, Invitrogen) or scr control with comparable gc-content (negative control high gc duplex, Invitrogen) using lipofectamin rnaimax transfection reagent (Invitrogen) according to manufacturer’s instructions.

### 4.8. SPARC Elisa Protein Measurement

Cell culture supernatant from F331 fibroblasts either transfected with stealth siRNA or scr control were collected at designated time points, aliquoted, flash frozen, and stored in liquid nitrogen. Next, samples were defrosted, centrifuged and the Quantikine SPARC ELISA (DSP00, R&D Systems, Minneapolis, MN, USA) carried out according to the manufacturer’s instructions.

### 4.9. Cancer Cell–Matrix Interactions

Cancer cells were seeded onto cell culture dishes coated with collagen I. In addition, either the collagen substrate or the culture medium was supplemented with 750 ng/mL rSPARC or equal amounts of PBS + 0.1% BSA as control. After 24 h incubation, cells were lysed, and proteins extracted for western blotting as described in [43]. Briefly, protein was extracted using HEPES lysis buffer supplemented with protease inhibitors cocktail (Complete, Roche, Germany) and phosphatase inhibitors. 10 µg protein per lane were analyzed by western blotting. The antibodies used recognized integrin β1 (1:1000; 4706, Cell Signaling Technology, Danvers, MA, USA), FAK (1:1000; 71,433, Cell Signaling Technology), p-FAKTyr397 (1:1000; 8556, Cell Signaling Technology), E-cadherin (1:1000; 3195, Cell Signaling Technology), and β-actin (1:5000, AC-15, Sigma, St. Louis, MO). Detection was achieved by chemiluminescence and band intensity was determined using ImageJ software. Uncropped scans of all blots can be found in Figure S5.

### 4.10. Migration Assay

For monitoring migration of cancer cells on collagen, black 96-well plates (Biozym Scientific, Oldendorf, Germany) were coated with 50 µg/mL collagen type I (Corning, Corning, NY, USA) supplemented with either 750 ng/mL human recombinant SPARC (rSPARC) (Peprotech) or equal volumes of 1×PBS + 0.1% BSA. 400 cancer cells stained with either CellTracker Orange or Green (Life Technologies, Carlsbad, CA, USA) were plated per well in 6 parallels per group. Migration was tracked every 15 min for a total of 22 h with appropriate filters using a live cell microscope (Nikon Eclipse Ti, Tokio, Japan) equipped with a humidified chamber supplemented with 7.5% CO_2_ at 37 °C. 

Migration of cancer cells on top of fibroblasts followed the same steps as mentioned above, but instead of collagen type I coating, F331 cells were used. 2 × 10^4^ fibroblasts either transfected with SPARC stealth siRNA or scr control were seeded into 96-well plates and were allowed to form dense monolayers with or without the presence of rSPARC for a total of 5 days. 

Evaluation of average mean velocity was performed for all cells tracked for a minimum of 10 frames using Fiji software [44]. Visualization of tracks was performed by using Chemotaxis and Migration Tool 2.0 software (Ibidi, Martinsried, Germany) and for better comparison, only tracks that were monitored for the full 22 h were used. In addition, to exclude artifacts due to floating cells, the first 4 frames were excluded from all migration experiments. Red circles were plotted as an indicator of track length and are based on 50% of the maximum Euclidian distance reached by HCT116 or SW480 migrating on F331 KD fibroblasts.

### 4.11. Organotypic Cell Culture Model

The basis of the collagen gels used was collagen type 1 (3 mg/mL, Corning) containing 10% 10× M199 medium (Sigma-Aldrich) and 0.22 M NaOH mixed with methylcellulose (Sigma-Aldrich) (MC) media (70% methylcellulose (1.5% *w/v* in DMEM and 2 mM I-glutamine), 20% DMEM and 10% FCS). 5*10^5^ fibroblasts were suspended in 500 µL consisting of equal amounts of collagen suspension and MC media and poured into 24-well plates. Depending on the experimental setup, rSPARC was added to the gels as well. Empty collagen gels were casted as controls. Collagen gels were allowed to solidify in a humidified chamber at 37 °C for 30 min and then supplied with 1 mL DMEM containing 10% FCS. After 4 days, 5 × 10^5^ cancer cells were added on top of both fibroblast containing as well as empty gels and allowed to attach for 24 h. Afterwards, gels were washed, supplied with DMEM containing 10% FCS and incubated at 37 °C in a humidified atmosphere with 7.5% CO_2_, renewing the media every 48 h. After additional 6 days, gels were washed with phosphate buffered saline (PBS), fixed with 1mL 4% formaldehyde (Histofix, PanReac AppliChem, Darmstadt, Germany) for 24 h and then processed for IHC.

### 4.12. Immunohistochemical Analysis

For every tissue reconstruct, 5 sections (4 µm thickness) each separated by 100 µm were analyzed to minimize the impact of potential outliers, as described in [31]. Slides were stained with monoclonal pan anti-cytokeratin antibody (C1801, Sigma-Aldrich) and SPARC antibody (D10F10, Cell Signaling) as described in [17]. 

For analysis, slides were digitalized using a microscopic slidescanner (Pannoramic Midi, 3DHistech, Budapest, Hungary) with a 40× objective. Invasion depth was measured by drawing straight lines with a 90° angle from the reconstruct border to the Pan-Cytokeratin positive cells inside the construct with Pannoramic Viewer software.

## 5. Conclusions

Taken together, the in vitro results demonstrate a pro-metastatic impact of stromal SPARC in CRC. This is in line with higher stromal SPARC abundance in stage II-IV CRC as compared to stage I [25] and with the association of high stromal SPARC with shorter survival in recurrent disease. As such, our study is the first that associates stromal SPARC with tumor progression and poor outcome in CRC based on both patient and in vitro data, which is well in line with recent reports from pancreatic, gastric and biliary cancer that all identify stromal SPARC as a predictor of worse survival [45,46,47]. For CRC, stromal SPARC seems to be a conditional predictor that only exerts a strong impact in recurrent disease. To evaluate this further, additional studies with larger cohorts of patients with rapid tumor progression will be necessary. Based on our results as well as reports that SPARC expression is related to therapy response in triple-negative breast cancer [48] and in CRC [29], SPARC could be useful as a predictive marker. We suggest investigating possible effects of SPARC levels towards treatment efficacy and clinical parameter with a focus on stage-dependent effects of SPARC abundance towards patient outcome.

## Figures and Tables

**Table 1:** Variables affecting 5-year DFS in the mRNA microarray cohort [18].

Variables	Univariate Cox Analysis			Multivariate Cox Analysis		
	HR	95% CI	*p*-Value	HR	95% CI	*p*-Value
Gender (male vs. female)	1.254	0.885–1.777	0.202			
Age	1.009	0.995–1.023	0.199			
**Staging (III vs. II)**	**1.958**	**1.388–2.764**	**<0.0001**	**1.836**	**1.221–3.761**	**0.003**
**Chemotherapy (yes vs. no)**	**1.526**	**1.085–2.146**	**0.015**	1.096	0.732–1.641	0.658
Location (proximal vs. distal)	0.877	0.618–1.246	0.464			
**SPARC (high vs. low)**	**1.410**	**1.001–1.989**	**0.050**	1.408	0.998–1.985	0.051

Variables affecting DFS were first analyzed in a univariate model. Those variables that had a statistically significant impact were then used in a multivariate Cox regression model. Significant variables are written in bold. DFS = disease-free survival; HR = hazard ratio; CI = confidence interval.

**Table 2:** Variables affecting 5-year OS in patients of the tissue microarray cohort with recurrent disease.

Variables	Univariate Cox Analysis			Multivariate Cox Analysis		
	HR	95% CI	*p*-Value	HR	95% CI	*p*-Value
Gender (male vs. female)	2.256	0.800–6.366	0.124			
**Age**	**1.052**	**1.001–1.105**	**0.045**	1.053	0.999–1.109	0.055
Staging (III vs. II)	1.907	0.550–6.608	0.309			
Chemotherapy (yes vs. no)	0.744	0.263–2.107	0.578			
**Location (proximal vs. distal)**	**2.362**	**0.932–5.992**	**0.070**	**0.338**	**0.123–0.931**	**0.036**
**SPARC (high vs. low)**	**3.607**	**1.337–9.732**	**0.011**	**4.574**	**1.609–13.004**	**0.004**

Variables affecting OS were first analyzed in a univariate model. Those variables that had a statistically significant impact were then analyzed together using multivariate Cox regression. Variables found to be significant are written in bold. OS = overall survival; HR = hazard ratio; CI = confidence interval.

## Supplementary Materials

The following are available online at https://www.mdpi.com/2072-6694/11/10/1421/s1, Figure S1: Overall-survival of CRC patients, Figure S2: Expression of common markers of used cells, Figure S3: Tissue reconstruct preparation, Figure S4: Invasion of SW480 cells, Figure S5: uncropped scans, Table S1: Patient clinical data and SPARC scores, Table S2: Recurrent Patient clinical data.
