# Supplementary material for: Impact of Fibroblast-Derived SPARC on Invasiveness of Colorectal Cancer Cells
Source: Cancers (Basel). 2019 Sep 24;11(10):1421. doi: 10.3390/cancers11101421 (PMC6827058; doi:10.3390/cancers11101421)
Supplement: Supplementary file 1 [file cancers-11-01421-s001.pdf]

Impact of Fibroblast-Derived SPARC on Invasiveness of Colorectal Cancer Cells

Daniel Drev, Felix Harpain, Andrea Beer, Anton Stift, Elisabeth S. Gruber, Martin Klimpfinger, Sabine Thalhammer, Andrea Reti, Lukas Kenner, Michael Bergmann and Brigitte Marian

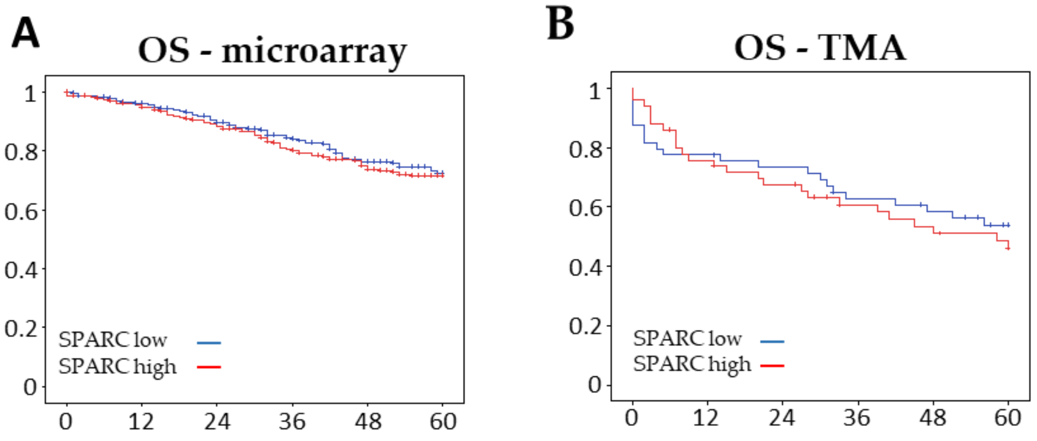

Figure S1. Overall-survival of CRC patients. (**A**) OS based on SPARC mRNA expression (n = 466). (**B**) SPARC abundance of tissue microarrays and OS (n = 85). Both Kaplan-Meier curves were plotted by using the median SPARC expression/abundance.

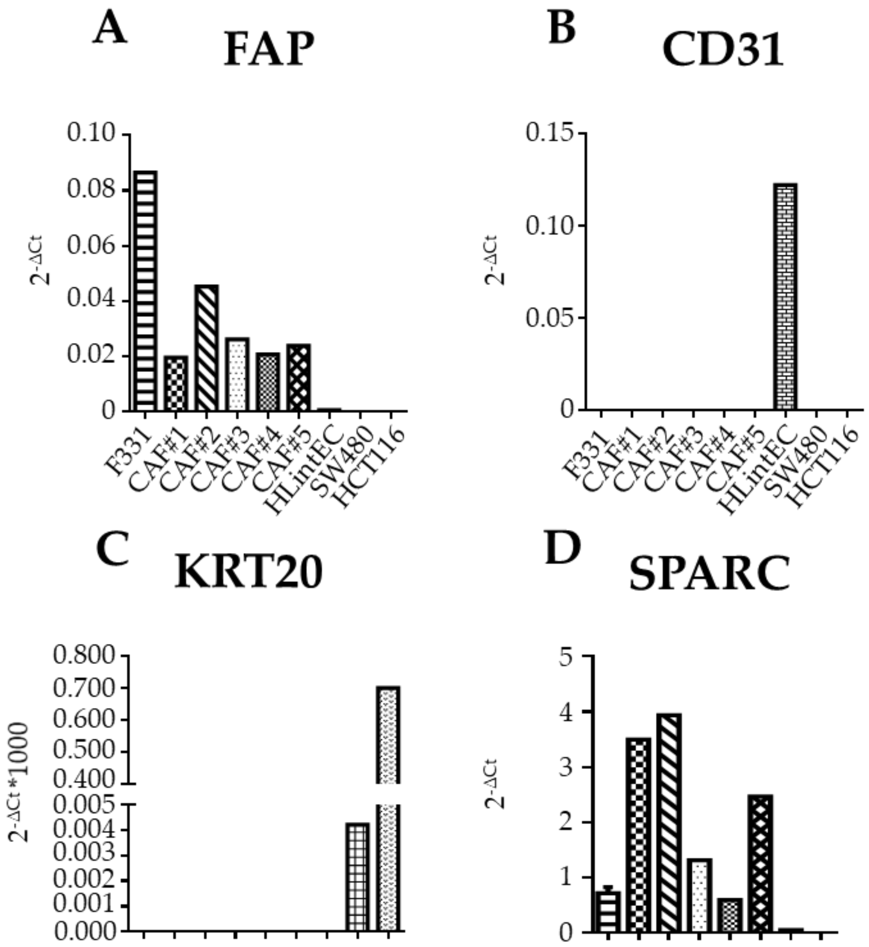

Figure S2. Expression of common markers of used cells**.** mRNA expression of the fibroblast activation protein alpha (FAP) (**A**) endothelial marker CD31 (**B**) colonic epithelial marker KRT20 (**C**) and SPARC (**D**) of various cells used. Human Large Intestine Microvascular Endothelial Cells (HLIntECs) were used as control.

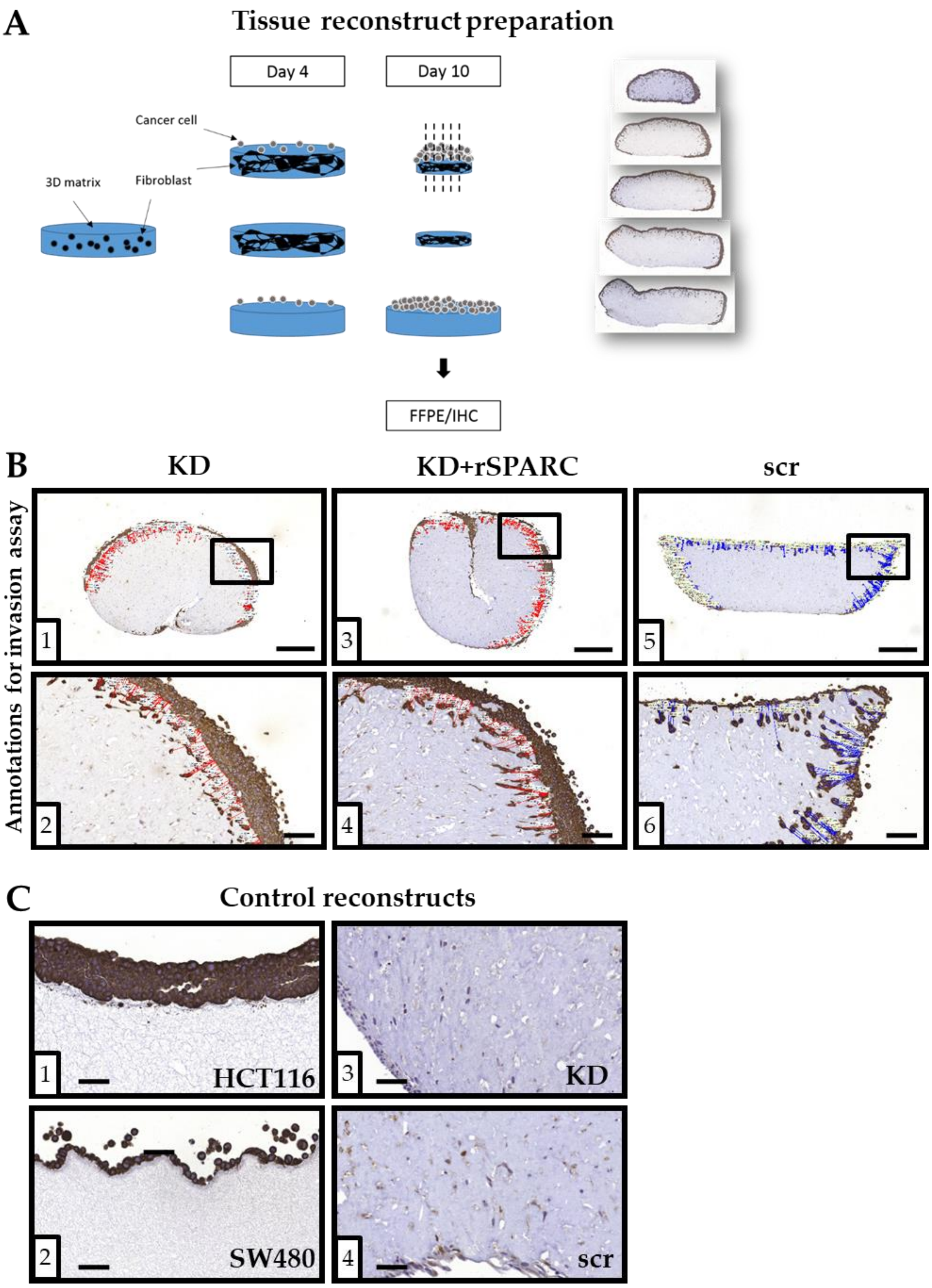

Figure S3. Tissue reconstruct preparation. Fibroblasts were suspended in collagen/methylcellulose and left for 4 days to structure the matrix before cancer cells were added. After 6 more days reconstructs were formalin-fixed, paraffin-embedded and sectioned, using five 4 µm sections separated by 100 µm for each reconstruct (**A**). Sections were pan-

cytokeratin stained to identify cancer cells. The invasion distance was measured as a straight line perpenticular to the border of the collagen substrate and the invading cancer cells (**B**). Size bars represent 500 μm in upper panels and 100μm in lower. HCT116 (**C**(1)) and SW480 (**C**(2)) were seeded on empty gels, incubated for 10 days and processed for IHC with pan-cytokeratin antibody. SPARC-depleted (**C**(3)) or scr control F331 (**C**(4)) reconstructs were IHC stained for SPARC protein on day 10; Size bar = 50 μm. IHC, immunohistochemistry; KD, knockdown; scr, scrambled.

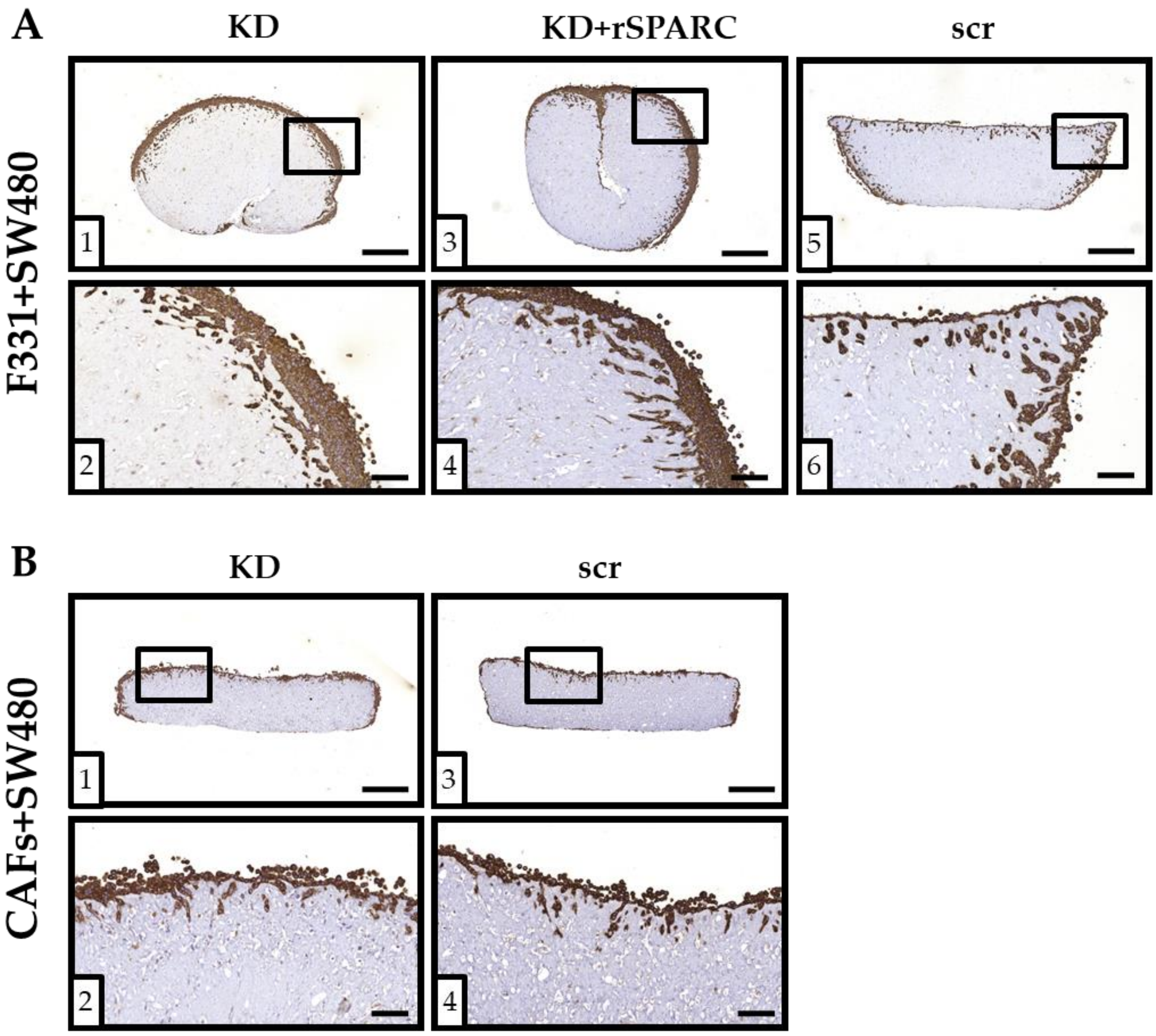

Figure S4. Invasion of SW480 cells. Typical examples of SW480 reconstruct sections co-cultured with either F331 (**A**) or CAFs (**B**) and IHC stained with an anti-cytokeratin antibody. Quantification is presented in Figure 6. Size bars represent 500 μm in upper and 100 μm in lower panels, respectively.

A

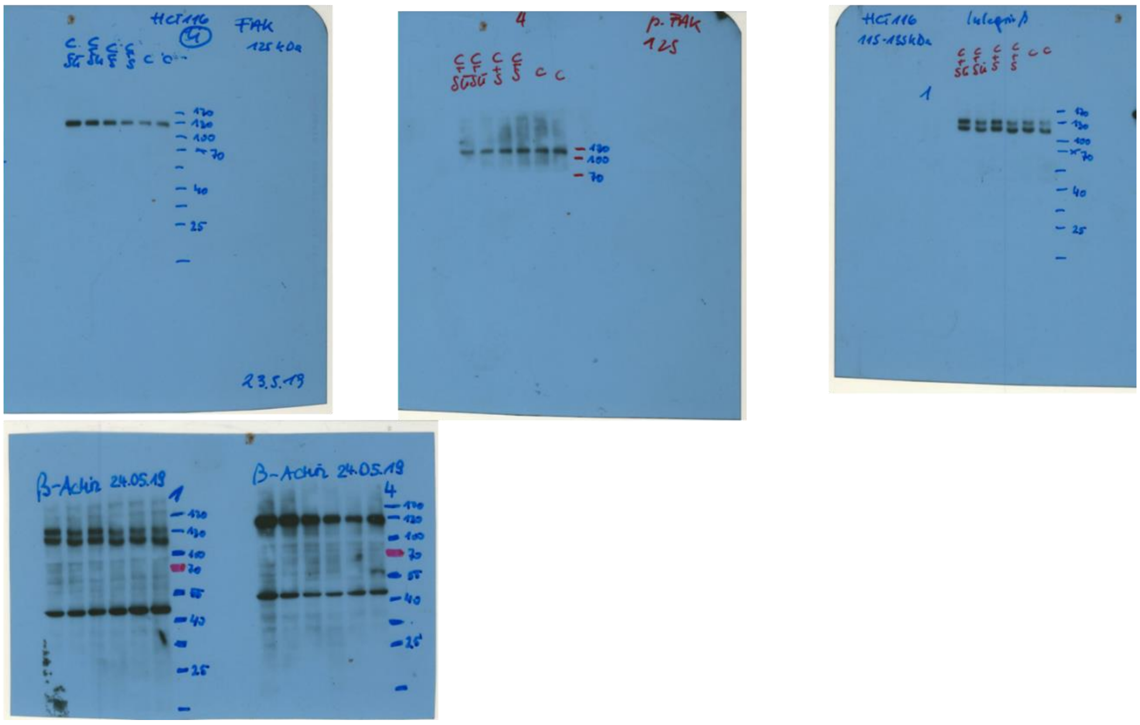
HCT116
FAK
125 kDa
23.5.19
p-FAK
125
β-Actin 24.05.19
B-Actin 24.05.19

B

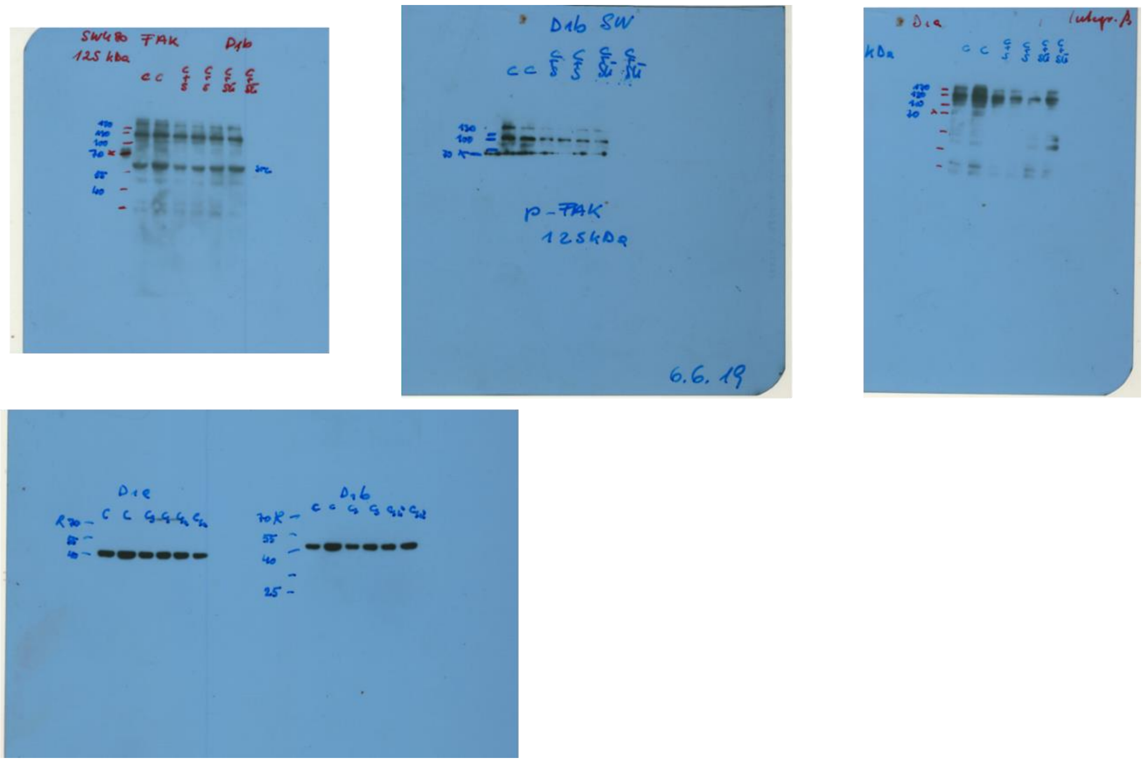
SW480 FAK
125 kDa
p-FAK
125kDa
6.6.19

C

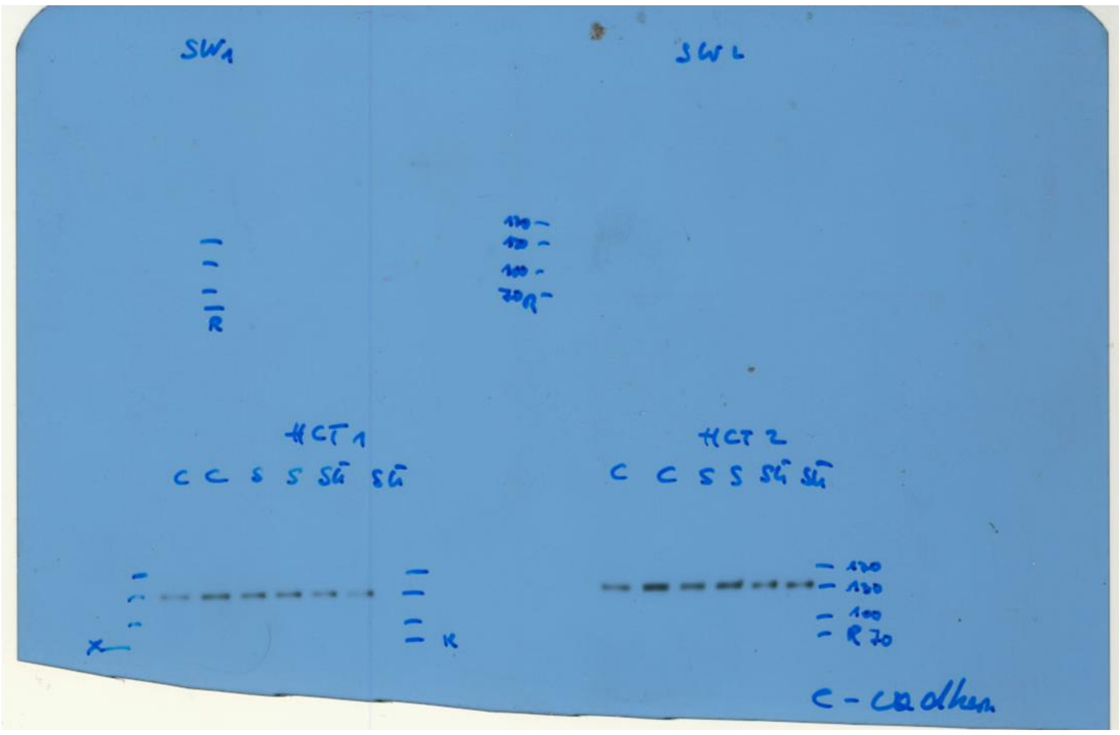

D

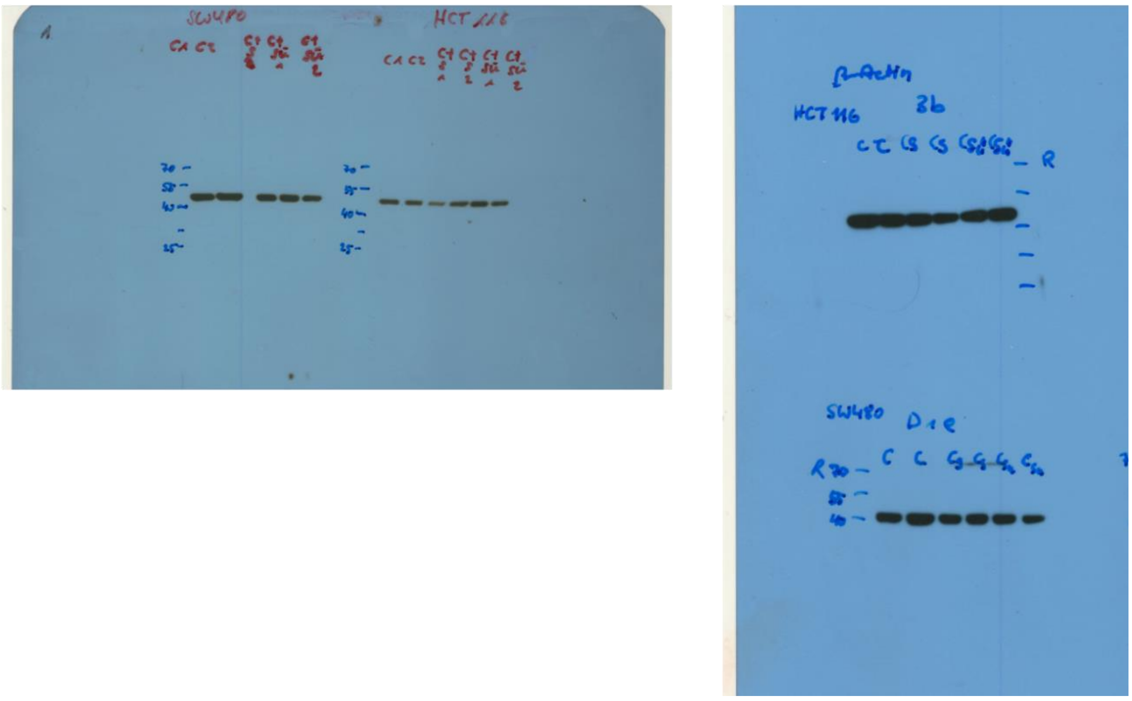

Figure S5. Uncropped scans. (**A**) Uncropped scans HCT116. Integrin β1 detected on blot 1; FAK & p-FAK on blot 4. (**B**). Uncropped scans SW480. Integrin β1 detected on blot 1a; FAK & p-FAK on blot 1b. β-actin detected on lower part of the blot. (**C**) Uncropped scans E-cadherin. Blots for both cell lines were exposed for the same time. (**D**) β-actin for the E-cadherin blots. For SW480 in experiment 1 one of the collagen/SPARC (Cs) groups was lost due to low protein concentration. Consequently, there were only 5 lanes on the blot. In experiment 2 there were 2 parallels for all experimental groups for both cell lines.

Table S1. Patient Clinical Data and SPARC Scores.

ID	Sex_f_m	age	Proximal_Distal	Staging	Ctx	Progr_Dis	Prog_Dis_Max12	Progr_Dis_Max60	RFS_Delay	RFS_Delay_Max12	RFS_Max60_delay	OS_Status_Max60	OS_Status	Osdelay	OSdelay_Max60	AverageSPARC	Epithl_S_Pos_n_y
1	0	57	1	3	1	1	1	1	6	6	6	1	1	45	45	50.8	1
2	1	85	1	2	0	0	0	0	2	2	2	1	1	2	2	20.8	0
3	1	87	0	2	0	0	0	0	0	0	0	1	1	0	0	45.0	0
4	1	72	1	3	1	0	0	0	66	12	60	0	0	66	60	65.0	0
5	0	66	1	2	0	0	0	0	46	12	46	0	0	46	46	36.7	0
6	1	78	0	2	1	0	0	0	15	12	15	1	1	15	15	45.0	0
7	0	57	1	2	0	0	0	0	60	12	60	0	0	60	60	69.2	0
8	0	69	0	3	1	0	0	0	3	3	3	1	1	3	3	77.5	0
9	1	58	0	2	0	0	0	0	33	12	33	0	0	33	33	63.3	0
10	1	73	1	3	1	1	0	1	25	12	25	0	0	68	60	36.7	0
11	1	74	0	3	0	1	0	1	37	12	37	1	1	41	41	50.8	1
12	0	70	0	2	0	0	0	0	20	12	20	1	1	20	20	76.3	0
13	1	79	1	3	1	0	0	0	2	2	2	1	1	2	2	79.2	0
14	1	68	0	3	1	0	0	0	32	12	32	0	0	32	32	34.2	0
15	1	59	1	2	1	1	0	1	46	12	46	0	0	64	60	15.0	1
16	1	76	1	3	1	1	0	0	87	12	60	0	0	101	60	55.0	0
17	1	90	0	3	1	1	0	1	30	12	30	1	1	42	42	16.3	0
18	0	77	1	3	0	0	0	0	31	12	31	0	0	31	31	60.0	0
19	1	67	1	3	1	1	1	1	1	1	1	1	1	28	28	24.2	0
20	1	78	1	3	1	0	0	0	67	12	60	0	0	67	60	4.5	1
21	1	88	0	3	1	0	0	0	72	12	60	0	1	72	60	56.7	0
22	0	86	0	2	0	0	0	0	0	0	0	1	1	0	0	33.8	0
23	1	80	1	3	0	0	0	0	29	12	29	0	0	29	29	60.0	0
24	0	55	1	3	1	0	0	0	74	12	60	0	0	74	60	46.3	0
25	0	95	0	3	0	0	0	0	13	12	13	0	0	13	13	9.5	0
26	0	93	0	3	0	0	0	0	68	12	60	0	0	68	60	65.0	0
27	1	74	1	3	0	0	0	0	3	3	3	1	1	3	3	86.7	0
28	1	77	1	3	1	0	0	0	135	12	60	0	1	135	60	15.8	1
29	1	82	0	2	1	0	0	0	63	12	60	0	1	63	60	32.5	0
30	1	82	1	3	1	0	0	0	7	7	7	1	1	7	7	45.0	1
31	0	90	0	3	1	0	0	0	70	12	60	0	1	70	60	60.0	0
32	0	76	0	3	1	0	0	0	2	2	2	1	1	2	2	27.5	0
33	1	73	0	2	0							0	0	13	13	53.3	0
34	0	80	0	3	1	1	1	1	6	6	6	1	1	7	7	58.3	0
35	1	61	0	2	0							0	0	6	6	66.7	0
36	0	65	0	2	1	1	0	1	44	12	44	0	1	69	60	35.0	0
37	1	66	0	3	1	1	1	1	11	11	11	1	1	13	13	55.0	0
38	1	62	1	2	0	1	1	1	5	5	5	1	1	27	27	57.5	0
39	0	89	0	3	1							1	1	14	14	10.0	0
40	0	93	1	3	1							1	1	47	47	35.8	0
41	1	83	0	3	1	1	1	1	3	3	3	1	1	3	3	50.0	0

42	0	93	1	3	0	0	0	0	61	12	60	0	1	61	60	44.2	0
43	1	76	1	3	0	0	0	0	0	0	0	1	1	0	0	48.3	0
44	1	88	1	2	0	1	1	1	4	4	4	1	1	28	28	53.3	0
45	0	88	1	3	0	1	0	1	16	12	16	1	1	34	34	22.5	0
46	1	69	0	3	1	0	0	0	82	12	60	0	0	82	60	19.6	0
47	1	69	1	3	0	0	0	0	26	12	26	0	0	26	26	53.3	1
48	0	63	1	3	1	0	0	0	76	12	60	0	0	76	60	65.8	0
49	0	77	1	3	1	1	1	1	11	11	11	1	1	32	32	16.3	0
50	1	79	1	2	0	0	0	0	39	12	39	1	1	39	39	49.2	0
51	1	67	0	2	0	1	0	1	13	12	13	1	1	56	56	20.8	0
52	0	54	1	2	1	0	0	0	84	12	60	0	0	84	60	5.3	0
53	1	67	0	3	1							1	1	5	5	36.7	0
54	0	68	1	3	1	1	0	1	41	12	41	0	0	88	60	50.0	0
55	0	62	1	3	1	0	0	0	78	12	60	0	0	78	60	60.0	0
56	0	71	1	3	1							1	1	0	0	17.5	0
57	0	58	0	3	0	1	0	1	60	12	60	0	1	72	60	37.5	0
58	1	72	1	3	1	1	1	1	6	6	6	1	1	8	8	46.7	0
59	0	78	1	2	0	0	0	0	57	12	57	0	0	57	57	27.5	0
60	0	85	0	2	0							0	0	90	60	24.6	0
61	1	62	1	2	1	1	0	0	78	12	60	0	0	81	60	10.0	0
62	1	83	0	3	1	1	1	1	4	4	4	1	1	9	9	49.2	0
63	0	61	1	3	1	1	0	1	17	12	17	1	1	60	60	45.0	0
64	1	75	0	2	0	0	0	0	49	12	49	0	0	49	49	51.7	0
65	0	75	1	3	1							0	1	85	60	30.0	1
66	1	73	0	2	0	0	0	0	2	2	2	1	1	2	2	25.0	0
67	0	70	1	3	1	1	0	1	17	12	17	0	0	59	59	35.0	0
68	1	96	0	3	1							1	1	20	20	18.3	1
69	0	74	1	3	1	1	0	0	91	12	60	0	0	173	60	30.0	0
70	1	73	0	3	1	1	1	1	2	2	2	1	1	5	5	60.8	0
71	1	81	1	2	0	0	0	0	90	12	60	0	0	90	60	37.5	0
72	1	90	0	2	0	0	0	0	21	12	21	1	1	21	21	46.7	0
73	1	86	1	3	1	0	0	0	4	4	4	1	1	4	4	18.3	1
74	0	91	0	2	0	0	0	0	66	12	60	0	1	66	60	36.7	0
75	0	62	1	2	1	0	0	0	94	12	60	0	0	94	60	25.8	0
76	0	78	0	2	0	0	0	0	33	12	33	1	1	33	33	75.0	0
77	1	82	1	2	0	0	0	0	53	12	53	0	0	53	53	36.8	0
78	1	77	0	3	1							1	1	58	58	45.0	1
79	1	66	0	3	1	1	1	1	6	6	6	1	1	7	7	68.3	0
80	1	72	1	3	1	1	0	1	22	12	22	1	1	51	51	9.7	1
81	0	82	1	3	0	0	0	0	70	12	60	0	0	70	60	62.5	0
82	1	69	1	2	0	0	0	0	93	12	60	0	1	93	60	30.0	0
83	1	78	0	2	0	0	0	0	0	0	0	1	1	0	0	13.3	0
84	1	67	0	2	0	0	0	0	102	12	60	0	0	102	60	60.0	1
85	1	88	1	2	0	0	0	0	97	12	60	0	0	97	60	54.2	0
86	0	97	0	3	1							0	0	83	60	36.7	1
87	0	92	0	2	1							1	1	0	0	9.7	0

88	1	70	1	2	0	0	0	0	31	12	31	1	1	31	31	40.0	1
89	0	90	0	2	0	0	0	0	0	0	0	1	1	0	0	33.3	0
90	1	67	1	3	1	0	0	0	55	12	55	0	0	55	55	10.0	0
91	1	88	0	3	1	0	0	0	100	12	60	0	0	100	60	49.2	0
92	1	69	1	3	0	0	0	0	100	12	60	0	0	100	60	55.8	0
93	1	85	1	2	0	0	0	0	99	12	60	0	0	99	60	76.7	0
94	0	87	1	3	0	0	0	0	30	12	30	1	1	30	30	27.5	0
95	1	77	0	3	1	0	0	0	48	12	48	1	1	48	48	51.3	0
96	1	91	1	2	0							0	0	86	60	58.3	0
97	0	71	1	3	0	0	0	0	0	0	0	1	1	0	0	33.3	1
98	1	70	0	2	0							0	0	107	60	64.2	0
99	1	74	0	2	0	0	0	0	102	12	60	0	0	102	60	32.5	0

Table S2. Recurrent Patient clinical data.

ID	location primary	Therapy Mode	Recurrence Location	Death
**2**	sigmoideum	Folfox/Bevazizumab; Folfiri/Bevazizumab; 5-FU/Bevazizumab	hepatic; pulmonary	1
**14**	sigmoideum	Folfoxiri/Bevazizumab; Folfox/Bevazizumab	hepatic	0
**15**	ascendens	XELOX/Bevazizumab	hepatic	1
**22**	sigmoideum	Folfox/Bevazizumab	pulmonary; osseous	0
**24**	rectum	radiation (50.4Gy)/Xeloda; Xelox/Bevazizumab	descendens; duodenal; peritoneal	0
**25**	ascendens	Xeloda	ascendens (Anastomose)	1
**28**	rectum	Xelox/Bevazizumab; Folfiri/Bevazizumab	hepatic; pulmonary	1
**46**	ascendens	none due to patient's death	hepatic	1
**48**	ascendens	Xeloda; Xelox after recurrence	lig. Rotundum	1
**50**	ascendens	Xelox	hepatic	1
**51**	sigmoideum	Xeloda/Bevazizumab; Cetuximab; Erbitux	hepatic	1
**55**	ascendens	Xelox	osseous	1
**60**	sigmoideum	Folfiri; Folfiri; Folfox/Bevazizumab	hepatic; pulmonary	1
**61**	sigmoideum	palliative therapy indicated; patient dies prior to implementation	pulmonary; mediastinal, osseous	1
**65**	sigmoideum	radiation therapy	hepatic; pelvic	1
**68**	descendens	Folfox; resection of lung metastasis	hepatic; pulmonary	1
**73**	sigmoideum	Folfox; Folfiri/Panitumab	hepatic	0
**78**	descendens	Folfox/Cetuximab; Folfiri/Cetuximab; Cetuximab mono	hepatic	1
**79**	sigmoideum	Bevazizumab; Irinotecan/Cetuximab	hepatic	1
**84**	sigmoideum	Xeloda; after HIPEC Xelox/Bevazizumab	peritoneal	0
**87**	ascendens	Xelox	hepatic	1
**89**	sigmoideum	Xelox/Radiation of hepatic lesion; Folfiri/Erbitux; Folfiri/Cetuximab	hepatic; pulmonary	1
**101**	rectum	Xelox, Xeliri/Bevazizumab; Folfiri/Cetuximab; Docetaxel; Xeloda/Bevazizumab	pulmonary	0
**108**	rectum	Irinotecan/5-FU; Xelox	ascendens, hepatic	0
**109**	ascendens	Irinotecan	hepatic; abdominal	1
**120**	ascendens	Xelox, terminated due to patient general condition	hepatic	1
**121**	sigmoideum	Xeloda/Oxaliplatin; Xeliri/Erbitux; Xelox/Bevazizumab; Sutent/Xeloda	peritoneal	1

HIPEC: hyperthermic intraperitoneale chemoperfusion.

© 2019 by the authors. Licensee MDPI, Basel, Switzerland. This article is an open access article distributed under the terms and conditions of the Creative Commons Attribution (CC BY) license (http://creativecommons.org/licenses/by/4.0/).
